# Poly[diaquadi-μ-hydroxido-κ^4^ *O*:*O*-dinitrato-κ^4^ *O*:*O*′-bis­[3-(pyridin-4-yl-κ*N*)-5-(pyridin-3-yl)-1,2,4-oxadiazole]dicopper(II)]

**DOI:** 10.1107/S1600536812010355

**Published:** 2012-03-14

**Authors:** Longfei Wu, Linxia Huang, Mouhai Shu

**Affiliations:** a School of Chemistry and Chemical Engineering, State Key Laboratory of Metal Matrix Composites, Shanghai Jiao Tong University, Shanghai 200240, People’s Republic of China

## Abstract

The title compound, [Cu_2_(NO_3_)_2_(OH)_2_(C_12_H_8_N_4_O)_2_(H_2_O)_2_]_*n*_, consists of a neutral polymeric Cu^II^ complex in which each Cu^II^ atom has a distorted octa­hedral geometry defined by a pyridyl N atom from a 3-(pyridin-3-yl)-5-(pyridin-4-yl)-1,2,4-oxadiazole ligand and five O atoms from a water mol­ecule, two nitrates and two hydroxides. Two Cu^II^ ions are bridged by two hydroxide anions resulting in a Cu_2_O_2_ loop, located across an inversion center and connected by the nitrate anions into a broad two-dimensional polymeric structure parallel to (100). In the crystal, there are O—H⋯O hydrogen bonds between the coodinated water mol­ecule and the nitrate and hydroxide, and between the hydroxide and the nitrate. Inter­molecular π–π inter­actions are present between pyridine rings in adjacent two-dimensional structures, with a centroid–centroid distance of 3.582 (2) Å.

## Related literature

For the preparation of the ligand, see: Chiou & Shine (1989 ▶). For a related structure, see: Sarkar *et al.* (2008 ▶).
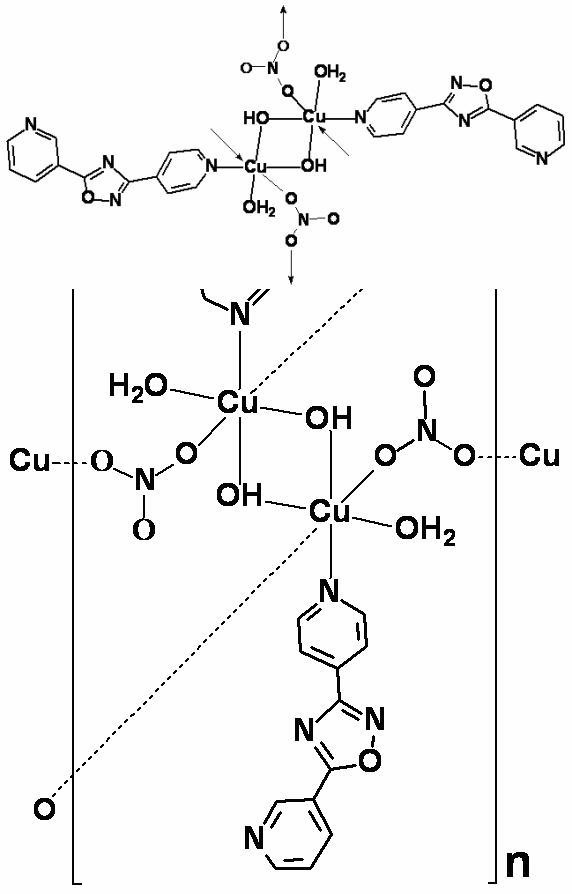


## Experimental

### 

#### Crystal data

[Cu_2_(NO_3_)_2_(OH)_2_(C_12_H_8_N_4_O)_2_(H_2_O)_2_]
*M* *_r_* = 384.81
Monoclinic, 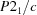

*a* = 17.7718 (11) Å
*b* = 5.9088 (4) Å
*c* = 13.6268 (8) Å
β = 105.572 (1)°
*V* = 1378.43 (15) Å^3^
*Z* = 4
Mo *K*α radiation
μ = 1.63 mm^−1^
*T* = 293 K
0.51 × 0.31 × 0.08 mm

#### Data collection

Bruker APEX CCD area-detector diffractometer
Absorption correction: multi-scan (*SADABS*; Bruker, 2000 ▶) *T*_min_ = 0.594, *T*_max_ = 1.000
7672 measured reflections
2976 independent reflections
2768 reflections with *I* > 2σ(*I*)
*R*_int_ = 0.119

#### Refinement

*R*[*F* ^2^ > 2σ(*F* ^2^)] = 0.048
*wR*(*F* ^2^) = 0.139
*S* = 1.05
2976 reflections
229 parameters
3 restraints
H atoms treated by a mixture of independent and constrained refinement
Δρ_max_ = 1.14 e Å^−3^
Δρ_min_ = −1.05 e Å^−3^

### 

Data collection: *SMART* (Bruker, 2000 ▶); cell refinement: *SAINT* (Bruker, 2000 ▶); data reduction: *SAINT*; program(s) used to solve structure: *SHELXTL* (Sheldrick, 2008 ▶); program(s) used to refine structure: *SHELXTL*; molecular graphics: *SHELXTL*; software used to prepare material for publication: *SHELXTL*, *publCIF* (Westrip, 2010 ▶) and *PLATON* (Spek, 2009 ▶).

## Supplementary Material

Crystal structure: contains datablock(s) I, global. DOI: 10.1107/S1600536812010355/bg2449sup1.cif

Structure factors: contains datablock(s) I. DOI: 10.1107/S1600536812010355/bg2449Isup2.hkl

Supplementary material file. DOI: 10.1107/S1600536812010355/bg2449Isup3.cdx

Additional supplementary materials:  crystallographic information; 3D view; checkCIF report

## Figures and Tables

**Table 1:** Hydrogen-bond geometry (Å, °)

*D*—H⋯*A*	*D*—H	H⋯*A*	*D*⋯*A*	*D*—H⋯*A*
O2—H6⋯O3^i^	0.86 (2)	1.84 (2)	2.680 (2)	166 (2)
O2—H7⋯O5^ii^	0.86 (3)	2.00 (3)	2.857 (3)	178 (5)
O2—H7⋯O6^ii^	0.86 (3)	2.58 (3)	3.137 (3)	124 (2)
O3—H8⋯O5^iii^	0.86 (2)	2.22 (3)	2.987 (3)	150 (3)

Symmetry codes: (i) 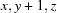; (ii) 
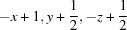
; (iii) 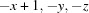.

**Fig. 1.**
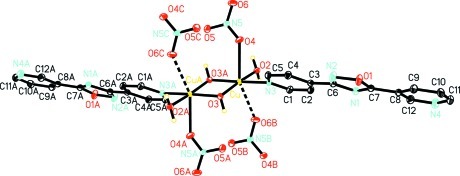
The molecular structure of the title complex with atom labels and 30% probability displacement ellipsoids for non-H atoms. Hydrogen atoms bound to the carbon atoms were omitted for clarity. Symmetry codes A: 1-x,-y,-z; B: x,1/2-y,-1/2+z; C: 1-x,-1/2+y,1/2-z.

**Fig. 2.**
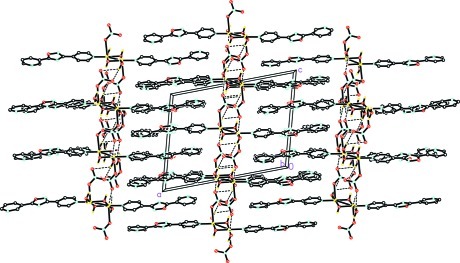
Packing view of the complex down the a axis, showing the two-dimensional structures in projection. O—H···O hydrogen bonds are shown in dashed lines. Note interdigitation of the aromatic groups in neighbouring two-dimensional structures, leading to π–π interactions.

## supplementary crystallographic information

### Comment

The title compound, [Cu_2_(C_12_H_8_N_4_O)_2_(H_2_O)_2_(OH)_2_ (NO_3_)_2_]_n_, was obtained unintentionally during an attempted synthesis of infinite network complex of copper(II) with a rod-like nitrogen containing ligand, 3-(pyridin-3-yl)-5-(pyridin-4-yl)-1,2,4-oxadiazole(Chiou and Shine, 1989).

In the structure, each Cu^II^ ion has a distorted octahedral geometry defined by a pyridyl N atom from a 3-(pyridin-3-yl)-5-(pyridin-4-yl)-1,2,4-oxadiazole ligand and five O atoms from a water molecule, two nitrates, and two hydroxides(Fig.1). For each Cu^II^ center, the equatorial sites are occupied by a water molecule, a pyridyl N atom, and two hydroxides, the apical sites being occupied by two oxygen atoms of nitrate anions. The equatorial bond distances are in the range 1.945 (2)-1.999 (2) Å while the axial bond distances are 2.431 (2) and 2.716 (2) Å. Neighbouring Cu^II^ ions are connected by two hydroxide anions into Cu_2_O_2_ loops with a Cu···Cu distance of 2.956 (1) Å. Similar Cu_2_O_2_ structure can be constructed by acetates (Sarkar *et al.* 2008). The result is the formation of centrosymmetric dimeric units, further connected by the nitrate anions to form broad two-dimensional structures parallel to (100).

In the crystal, there are O—H···O hydrogen bonds between the coodinated water molecule and the nitrate or hydroxide, and between the hydroxide and the nitrate. The details are listed in Table 1. There are also intermolecular π—π interactions between pyridine rings in neighboring two-dimensional structures, with a centroid—centroid distance of 3.582 (2)Å (Fig 2).

### Experimental

A solution of 3-(pyridin-3-yl)-5-(pyridin-4-yl)-1,2,4-oxadiazole(0.2 mmol) in methanol(10 ml) was layered carefully on the solution of Cu(NO_3_)_2_.3H_2_O(0.2 mmol) in water(5 ml) in a straight test tube. The test tube was covered and kept in the refrigerator. Green crystals suitable for X-ray diffraction analysis were obtained after 2 weeks.

### Refinement

H atoms bonded to O atoms were located in a difference map and refined with distance restraints of O—H = 0.86 (2) Å. Other H atoms were positioned geometrically and refined using a riding model with C—H = 0.93(aromatic) and C—H = 0.96(CH_3_) Å. All H atoms were refined with *U*_iso_(H) = 1.2 (1.5 for methyl groups) times*U*_eq_(C).

### Crystal data

**Table:**

[Cu_2_(NO_3_)_2_(OH)_2_(C_12_H_8_N_4_O)_2_(H_2_O)_2_]	*F*(000) = 780
*M**_r_* = 384.81	*D*_x_ = 1.854 Mg m^−^^3^
Monoclinic, *P*2_1_/*c*	Mo *K*α radiation, λ = 0.71073 Å
Hall symbol: -P 2ybc	Cell parameters from 5190 reflections
*a* = 17.7718 (11) Å	θ = 4.8–56.4°
*b* = 5.9088 (4) Å	µ = 1.63 mm^−^^1^
*c* = 13.6268 (8) Å	*T* = 293 K
β = 105.572 (1)°	Block, blue
*V* = 1378.43 (15) Å^3^	0.51 × 0.31 × 0.08 mm
*Z* = 4	

### Data collection

**Table:**

Bruker APEX CCD area-detector diffractometer	2976 independent reflections
Radiation source: fine-focus sealed tube	2768 reflections with *I* > 2σ(*I*)
Graphite monochromator	*R*_int_ = 0.119
phi and ω scans	θ_max_ = 27.0°, θ_min_ = 1.2°
Absorption correction: multi-scan (*SADABS*; Bruker, 2000)	*h* = −17→22
*T*_min_ = 0.594, *T*_max_ = 1.000	*k* = −7→7
7672 measured reflections	*l* = −17→17

### Refinement

**Table:**

Refinement on *F*^2^	Secondary atom site location: difference Fourier map
Least-squares matrix: full	Hydrogen site location: inferred from neighbouring sites
*R*[*F*^2^ > 2σ(*F*^2^)] = 0.048	H atoms treated by a mixture of independent and constrained refinement
*wR*(*F*^2^) = 0.139	*w* = 1/[σ^2^(*F*_o_^2^) + (0.089*P*)^2^] where *P* = (*F*_o_^2^ + 2*F*_c_^2^)/3
*S* = 1.05	(Δ/σ)_max_ = 0.001
2976 reflections	Δρ_max_ = 1.14 e Å^−^^3^
229 parameters	Δρ_min_ = −1.05 e Å^−^^3^
3 restraints	Extinction correction: *SHELXTL* (Sheldrick, 2008), Fc^*^=kFc[1+0.001xFc^2^λ^3^/sin(2θ)]^-1/4^
Primary atom site location: structure-invariant direct methods	Extinction coefficient: 0.0143 (18)

### Special details

**Table:**

Geometry. All e.s.d.'s (except the e.s.d. in the dihedral angle between two l.s. planes) are estimated using the full covariance matrix. The cell e.s.d.'s are taken into account individually in the estimation of e.s.d.'s in distances, angles and torsion angles; correlations between e.s.d.'s in cell parameters are only used when they are defined by crystal symmetry. An approximate (isotropic) treatment of cell e.s.d.'s is used for estimating e.s.d.'s involving l.s. planes.

### Fractional atomic coordinates and isotropic or equivalent isotropic displacement parameters (Å^2^)

**Table:**

	*x*	*y*	*z*	*U*_iso_*/*U*_eq_	
Cu	0.568231 (15)	0.14812 (5)	0.04093 (2)	0.02002 (18)	
O1	1.03390 (11)	−0.2157 (4)	0.16936 (16)	0.0378 (5)	
O2	0.58494 (10)	0.4687 (3)	0.08870 (12)	0.0228 (4)	
O3	0.54446 (11)	−0.1523 (3)	−0.02081 (14)	0.0211 (4)	
O4	0.59173 (11)	0.0905 (4)	0.22324 (14)	0.0351 (4)	
O5	0.47452 (11)	0.0938 (4)	0.24387 (14)	0.0327 (4)	
O6	0.57569 (13)	0.1084 (4)	0.37477 (14)	0.0433 (5)	
N1	0.98046 (13)	0.1098 (4)	0.10703 (16)	0.0270 (5)	
N2	0.95355 (13)	−0.2348 (5)	0.1616 (2)	0.0391 (6)	
N3	0.68350 (13)	0.1057 (4)	0.06699 (16)	0.0219 (4)	
N4	1.20685 (16)	0.3615 (4)	0.1113 (2)	0.0412 (6)	
N5	0.54702 (13)	0.0982 (4)	0.28059 (16)	0.0268 (5)	
C1	0.73297 (14)	0.2588 (4)	0.04712 (19)	0.0262 (5)	
H1B	0.7129	0.3968	0.0190	0.031*	
C2	0.81232 (15)	0.2217 (5)	0.06637 (19)	0.0274 (5)	
H2C	0.8449	0.3335	0.0528	0.033*	
C3	0.84262 (14)	0.0141 (4)	0.10637 (17)	0.0229 (5)	
C4	0.79131 (16)	−0.1452 (4)	0.1275 (2)	0.0278 (6)	
H4B	0.8097	−0.2849	0.1551	0.033*	
C5	0.71365 (14)	−0.0937 (5)	0.10707 (19)	0.0260 (5)	
H5A	0.6800	−0.2015	0.1215	0.031*	
C6	0.92614 (14)	−0.0393 (4)	0.12543 (18)	0.0246 (5)	
C7	1.04446 (14)	−0.0069 (4)	0.13676 (18)	0.0255 (5)	
C8	1.12382 (14)	0.0631 (5)	0.13945 (18)	0.0252 (5)	
C9	1.18772 (15)	−0.0806 (5)	0.1720 (2)	0.0304 (5)	
H9A	1.1814	−0.2285	0.1918	0.036*	
C10	1.26094 (16)	0.0024 (5)	0.1741 (2)	0.0355 (6)	
H10A	1.3049	−0.0888	0.1957	0.043*	
C11	1.26778 (16)	0.2211 (6)	0.1440 (2)	0.0377 (7)	
H11A	1.3174	0.2750	0.1464	0.045*	
C12	1.13696 (16)	0.2822 (5)	0.1103 (2)	0.0334 (6)	
H12A	1.0942	0.3783	0.0889	0.040*	
H7	0.5678 (18)	0.504 (6)	0.1398 (16)	0.045 (9)*	
H8	0.5559 (17)	−0.160 (4)	−0.0774 (13)	0.024 (5)*	
H6	0.5725 (15)	0.576 (4)	0.0449 (17)	0.024 (5)*	

### Atomic displacement parameters (Å^2^)

**Table:**

	*U*^11^	*U*^22^	*U*^33^	*U*^12^	*U*^13^	*U*^23^
Cu	0.0140 (2)	0.0237 (3)	0.0225 (2)	−0.00041 (9)	0.00511 (15)	−0.00202 (9)
O1	0.0176 (9)	0.0423 (11)	0.0543 (12)	0.0047 (8)	0.0112 (8)	0.0142 (10)
O2	0.0233 (8)	0.0238 (9)	0.0227 (8)	−0.0004 (7)	0.0087 (7)	−0.0011 (7)
O3	0.0163 (9)	0.0265 (10)	0.0214 (8)	0.0014 (6)	0.0066 (7)	−0.0020 (6)
O4	0.0307 (10)	0.0526 (11)	0.0257 (9)	0.0025 (9)	0.0141 (8)	0.0074 (9)
O5	0.0248 (10)	0.0409 (11)	0.0336 (10)	−0.0020 (8)	0.0098 (7)	0.0022 (8)
O6	0.0442 (12)	0.0669 (14)	0.0187 (9)	0.0024 (10)	0.0083 (8)	0.0033 (9)
N1	0.0185 (10)	0.0351 (11)	0.0282 (11)	0.0013 (9)	0.0077 (8)	0.0006 (9)
N2	0.0186 (11)	0.0451 (15)	0.0544 (15)	0.0036 (11)	0.0114 (10)	0.0152 (12)
N3	0.0151 (10)	0.0285 (10)	0.0223 (10)	0.0001 (8)	0.0051 (8)	−0.0007 (8)
N4	0.0341 (15)	0.0455 (16)	0.0456 (15)	−0.0060 (10)	0.0133 (11)	0.0039 (11)
N5	0.0292 (12)	0.0311 (10)	0.0223 (10)	−0.0024 (10)	0.0106 (8)	0.0038 (9)
C1	0.0225 (13)	0.0269 (12)	0.0309 (12)	0.0006 (10)	0.0102 (9)	0.0037 (10)
C2	0.0212 (12)	0.0317 (13)	0.0309 (12)	−0.0018 (11)	0.0098 (10)	0.0028 (11)
C3	0.0185 (11)	0.0316 (12)	0.0190 (10)	−0.0007 (9)	0.0057 (8)	−0.0005 (9)
C4	0.0216 (14)	0.0303 (14)	0.0307 (13)	0.0007 (9)	0.0053 (10)	0.0072 (9)
C5	0.0197 (12)	0.0289 (12)	0.0287 (12)	−0.0025 (10)	0.0053 (9)	0.0048 (10)
C6	0.0185 (11)	0.0340 (14)	0.0218 (11)	0.0004 (10)	0.0062 (9)	−0.0002 (9)
C7	0.0189 (11)	0.0360 (13)	0.0217 (10)	0.0028 (10)	0.0058 (9)	−0.0004 (10)
C8	0.0174 (11)	0.0363 (14)	0.0226 (11)	0.0019 (10)	0.0065 (8)	−0.0019 (10)
C9	0.0231 (13)	0.0359 (14)	0.0328 (13)	0.0025 (11)	0.0086 (10)	−0.0006 (11)
C10	0.0186 (12)	0.0509 (17)	0.0354 (13)	0.0047 (12)	0.0046 (10)	−0.0072 (13)
C11	0.0220 (13)	0.0542 (18)	0.0387 (15)	−0.0071 (13)	0.0110 (11)	−0.0059 (14)
C12	0.0247 (13)	0.0404 (15)	0.0351 (13)	0.0035 (12)	0.0082 (10)	0.0034 (12)

### Geometric parameters (Å, º)

**Table:**

Cu—O3^i^	1.9474 (19)	N4—C11	1.342 (4)
Cu—O3	1.9613 (16)	C1—C2	1.381 (3)
Cu—N3	1.999 (2)	C1—H1B	0.9300
Cu—O2	1.9992 (17)	C2—C3	1.390 (4)
Cu—O4	2.4301 (18)	C2—H2C	0.9300
Cu—Cu^i^	2.9559 (5)	C3—C4	1.393 (4)
O1—C7	1.341 (3)	C3—C6	1.471 (3)
O1—N2	1.408 (3)	C4—C5	1.367 (4)
O2—H7	0.856 (10)	C4—H4B	0.9300
O2—H6	0.859 (10)	C5—H5A	0.9300
O3—H8	0.849 (10)	C7—C8	1.461 (3)
O4—N5	1.256 (3)	C8—C12	1.392 (4)
O5—N5	1.251 (3)	C8—C9	1.392 (4)
O6—N5	1.249 (3)	C9—C10	1.384 (4)
N1—C7	1.299 (3)	C9—H9A	0.9300
N1—C6	1.379 (3)	C10—C11	1.371 (4)
N2—C6	1.298 (4)	C10—H10A	0.9300
N3—C1	1.339 (3)	C11—H11A	0.9300
N3—C5	1.348 (3)	C12—H12A	0.9300
N4—C12	1.324 (4)		
			
O3^i^—Cu—O3	81.73 (7)	C2—C1—H1B	118.4
O3^i^—Cu—N3	173.18 (8)	C1—C2—C3	118.9 (2)
O3—Cu—N3	93.16 (8)	C1—C2—H2C	120.6
O3^i^—Cu—O2	95.23 (7)	C3—C2—H2C	120.6
O3—Cu—O2	173.40 (7)	C2—C3—C4	118.1 (2)
N3—Cu—O2	90.31 (8)	C2—C3—C6	121.7 (2)
O3^i^—Cu—O4	91.93 (7)	C4—C3—C6	120.3 (2)
O3—Cu—O4	105.65 (8)	C5—C4—C3	119.3 (2)
N3—Cu—O4	85.07 (7)	C5—C4—H4B	120.4
O2—Cu—O4	80.23 (7)	C3—C4—H4B	120.4
O3^i^—Cu—Cu^i^	41.04 (5)	N3—C5—C4	123.1 (2)
O3—Cu—Cu^i^	40.69 (6)	N3—C5—H5A	118.4
N3—Cu—Cu^i^	133.69 (6)	C4—C5—H5A	118.4
O2—Cu—Cu^i^	135.98 (5)	N2—C6—N1	115.3 (2)
O4—Cu—Cu^i^	101.61 (5)	N2—C6—C3	121.1 (2)
C7—O1—N2	106.1 (2)	N1—C6—C3	123.6 (2)
Cu—O2—H7	116 (2)	N1—C7—O1	113.8 (2)
Cu—O2—H6	119 (2)	N1—C7—C8	128.0 (2)
H7—O2—H6	108 (3)	O1—C7—C8	118.2 (2)
Cu^i^—O3—Cu	98.27 (7)	C12—C8—C9	118.3 (2)
Cu^i^—O3—H8	111 (2)	C12—C8—C7	119.4 (2)
Cu—O3—H8	111.2 (17)	C9—C8—C7	122.3 (2)
N5—O4—Cu	131.85 (16)	C10—C9—C8	118.2 (3)
C7—N1—C6	101.6 (2)	C10—C9—H9A	120.9
C6—N2—O1	103.1 (2)	C8—C9—H9A	120.9
C1—N3—C5	117.5 (2)	C11—C10—C9	119.1 (3)
C1—N3—Cu	125.22 (18)	C11—C10—H10A	120.4
C5—N3—Cu	117.30 (17)	C9—C10—H10A	120.4
C12—N4—C11	117.1 (3)	N4—C11—C10	123.6 (3)
O6—N5—O5	120.3 (2)	N4—C11—H11A	118.2
O6—N5—O4	119.3 (2)	C10—C11—H11A	118.2
O5—N5—O4	120.4 (2)	N4—C12—C8	123.7 (3)
N3—C1—C2	123.1 (2)	N4—C12—H12A	118.1
N3—C1—H1B	118.4	C8—C12—H12A	118.1
			
O3^i^—Cu—O3—Cu^i^	0.0	Cu—N3—C5—C4	179.3 (2)
N3—Cu—O3—Cu^i^	175.46 (7)	C3—C4—C5—N3	0.0 (4)
O4—Cu—O3—Cu^i^	89.69 (8)	O1—N2—C6—N1	0.6 (3)
O3^i^—Cu—O4—N5	−9.7 (2)	O1—N2—C6—C3	−179.3 (2)
O3—Cu—O4—N5	−91.7 (3)	C7—N1—C6—N2	−1.3 (3)
N3—Cu—O4—N5	176.4 (3)	C7—N1—C6—C3	178.6 (2)
O2—Cu—O4—N5	85.3 (2)	C2—C3—C6—N2	−179.1 (3)
Cu^i^—Cu—O4—N5	−49.9 (2)	C4—C3—C6—N2	0.3 (4)
C7—O1—N2—C6	0.3 (3)	C2—C3—C6—N1	1.0 (4)
O2—Cu—N3—C1	−40.0 (2)	C4—C3—C6—N1	−179.6 (2)
O4—Cu—N3—C1	−120.2 (2)	C6—N1—C7—O1	1.5 (3)
Cu^i^—Cu—N3—C1	138.47 (18)	C6—N1—C7—C8	−177.8 (2)
O3—Cu—N3—C5	−45.1 (2)	N2—O1—C7—N1	−1.2 (3)
O2—Cu—N3—C5	140.52 (18)	N2—O1—C7—C8	178.1 (2)
O4—Cu—N3—C5	60.36 (18)	N1—C7—C8—C12	2.0 (4)
Cu^i^—Cu—N3—C5	−41.0 (2)	O1—C7—C8—C12	−177.2 (2)
Cu—O4—N5—O6	−165.23 (19)	N1—C7—C8—C9	−179.1 (3)
Cu—O4—N5—O5	15.4 (4)	O1—C7—C8—C9	1.7 (4)
C5—N3—C1—C2	−0.4 (4)	C12—C8—C9—C10	0.3 (4)
Cu—N3—C1—C2	−179.92 (19)	C7—C8—C9—C10	−178.5 (2)
N3—C1—C2—C3	1.4 (4)	C8—C9—C10—C11	−0.2 (4)
C1—C2—C3—C4	−1.5 (4)	C12—N4—C11—C10	1.2 (4)
C1—C2—C3—C6	177.9 (2)	C9—C10—C11—N4	−0.5 (4)
C2—C3—C4—C5	0.9 (4)	C11—N4—C12—C8	−1.1 (4)
C6—C3—C4—C5	−178.6 (2)	C9—C8—C12—N4	0.3 (4)
C1—N3—C5—C4	−0.3 (4)	C7—C8—C12—N4	179.2 (3)

Symmetry code: (i) −*x*+1, −*y*, −*z*.

### Hydrogen-bond geometry (Å, º)

**Table:**

*D*—H···*A*	*D*—H	H···*A*	*D*···*A*	*D*—H···*A*
O2—H6···O3^ii^	0.86 (2)	1.84 (2)	2.680 (2)	166 (2)
O2—H7···O5^iii^	0.86 (3)	2.00 (3)	2.857 (3)	178 (5)
O2—H7···O6^iii^	0.86 (3)	2.58 (3)	3.137 (3)	124 (2)
O3—H8···O5^i^	0.86 (2)	2.22 (3)	2.987 (3)	150 (3)

Symmetry codes: (i) −*x*+1, −*y*, −*z*; (ii) *x*, *y*+1, *z*; (iii) −*x*+1, *y*+1/2, −*z*+1/2.
